# A Cross-Sectional Survey of Knowledge of Skin Cancer in Saudi Arabia

**DOI:** 10.5826/dpc.1103a76

**Published:** 2021-05-20

**Authors:** Hend M. Al-Atif

**Affiliations:** 1 Department of Internal Medicine, College of Medicine, King Khalid University, Abha, Saudi Arabia

**Keywords:** Skin cancer, sunscreen, sunburn, sun exposure, questionnaire, sun-protective behavior

## Abstract

**Background:**

Skin cancer has become one of the world’s leading health problems, and incidence rates are on the rise. The leading causes of skin cancer are sun exposure, family history and sunburn, and the most agreed-upon preventative behaviors are sunscreen application and sun avoidance.

**Objectives:**

This study assessed the knowledge of the causes of skin cancer and awareness of preventative measures in Saudi Arabia.

**Methods:**

A cross-sectional study was conducted among 529 participants in a WhatsApp group over 3 months. Consenting participants completed a validated, 18-item questionnaire.

**Results:**

Of 529 total participants, nearly 55% of participants reported an awareness of skin cancer, 35% understood its metastasis and 55.1% knew about its spread. However, 44% of participants were unaware of the different types of the disease. Social media was reported to be the most common source of information. The majority of participants were able to identify symptoms of skin cancer and had knowledge of risk factors. Most participants understood proper preventative measures, and reported that they use sunscreen regularly.

**Conclusions:**

The general knowledge of skin cancer in Saudi Arabia is not high but is increasing. However, sun-protective behaviors are lacking, despite the population’s knowledge of the benefits. Awareness campaigns and incentive programs may encourage better preventative behavior. Future studies should explore participants’ awareness of more specific aspects of skin cancer using a more diverse and extensive population sample.

## Introduction

Skin cancer is a group of diseases characterized by the abnormal mutation and multiplication of cells in the epidermal layer of the skin [1]. It is a major health problem worldwide, and has a great economic burden in both developed and developing nations [2]. There are two main types of skin cancer: melanoma, which is more likely to result in death, and non-melanoma skin cancer, which includes basal cell carcinoma and squamous cell carcinoma [3]. Skin cancer is much more common among white populations than among people of color [4], although fewer data are available on skin cancer in non-white populations [5]. The worldwide prevalence of skin cancer is 197.9 per 100,000 [6], with the highest rates occurring in Australia and New Zealand [6,7]. The global incidence of skin cancer is increasing [8]. However, this finding may be due to an increase in screenings and biopsies, as opposed to an increase in actual disease prevalence [9].

The most-cited cause of skin cancer is exposure to sunlight, specifically UV irradiation [10], which has been exacerbated by depletion of the ozone layer. Therefore, living in sunny climates is a risk factor [11]. Other risk factors include a family history of skin cancer, a history of severe sunburn and the age at which sunburns occur [12], as well as having fair skin [13]. A survey by Al-Dawsari and Shahab [14], conducted in 2016 in the eastern part of Saudi Arabia, found that 60% of subjects described their skin as being of the Fitzpatrick type III or V while the rest described their skin as types I or II. A cross-sectional study by Alliali et al in Makkah, Saudi Arabia, in 2014 found that almost two-thirds of Saudi women had a naturally light type of skin (types III or IV) [15].

The main appearance of basal cell carcinoma on the skin is a pigmented lesion, which can be pink, brown or black-blue, while squamous cell carcinoma appears as a rough or scaly, red-brown papule [16]. The most commonly used approach for diagnosing irregular moles and pigmented lesions as melanoma is the ABCDE method, which stands for “Asymmetry, Border irregularity, Color variation, Diameter and Evolving” [11]. Symptoms of advanced melanoma include anorexia, bleeding, pain, and fatigue, among others [17].

Skin cancer awareness increases with the incidence rate in a population. Several studies have shown a high level of general awareness of skin cancer globally, including in the United Kingdom and Pakistan [18,19]. One systematic review of North American studies found that skin cancer knowledge had a positive association with sun-protective behavior [20]. One study found that women have a greater awareness of skin cancer than men [21]. Furthermore, one US study showed higher levels of awareness among white Americans than Hispanic or African-American participants [22].

A study conducted in Riyadh, Saudi Arabia, showed that the population had a good general awareness of skin cancer but little awareness of specific details of the disease, with 83% stating they were unaware that sun exposure causes skin cancer [23]. However, a later study in Jeddah, Saudi Arabia, showed that 73% of participants were aware of the association between skin cancer and exposure to sunlight, although 58% did not know that moderate tanning also poses a risk [24]. This study assessed the knowledge of skin cancer in the Saudi Arabia population through a questionnaire designed to measure this knowledge more accurately than in previous studies.

## Methods

### Study Design

A population-based, cross-sectional survey was conducted over a period of 3 months, beginning in January 2020. People from the general population of Saudi Arabia were eligible to participate if they were ≥18 years old. They were informed that the data were to be collected anonymously, and were asked to provide consent before responding. Anonymous reporting and confidentiality protocols were maintained at all levels. Ethical approval was obtained from King Khalid University Review Board. A WhatsApp group was created for distributing the study form, which consisted of an 18-item questionnaire, designed on Google Forms.

### Data Collection

This study used a validated questionnaire that was developed on the basis of an in-depth literature search for surveys of skin cancer awareness conducted in different countries. Questions from previously published [25,26] studies were adapted on the basis of feedback from 3 experts in skin disease research. These previously published studies included one by Al Robaee [25] and one by Agarwal et al [26]. The questionnaire was piloted with a separate random sample of 30 participants, who provided feedback on the clarity of questions.

The questionnaire collected sociodemographic data, including age, gender and education level. Questions were designed to assess participants’ general knowledge of skin cancer, hazards of excessive sunlight exposure, protection methods and sunscreen use. General knowledge of skin cancer was measured by asking about the participants’ history of skin cancer, the sources of their information, and the appearance of, risk factors for and prevention of skin cancer. Questions were asked about the participants’ knowledge of the pros and cons of using sunscreen, on what basis they select a sunscreen, the site and frequency of usage, and any complications after sunscreen application.

### Statistical Analysis

Data entry and statistical analysis were performed using Statistical Package for Social Sciences software v25 (SPSS). Continuous variables are presented as mean and standard deviation (SD), and categorical variables are presented as numbers and percentages.

## Results

Data were collected from 529 participants with a mean age of 36 years (SD = 10 years). The majority of participants were women and university educated (Table 1). About 55.1% of participants knew about skin cancer (n=285), primarily from social media. Knowledge of specific types of skin cancer was lower.

**Figure 1:**
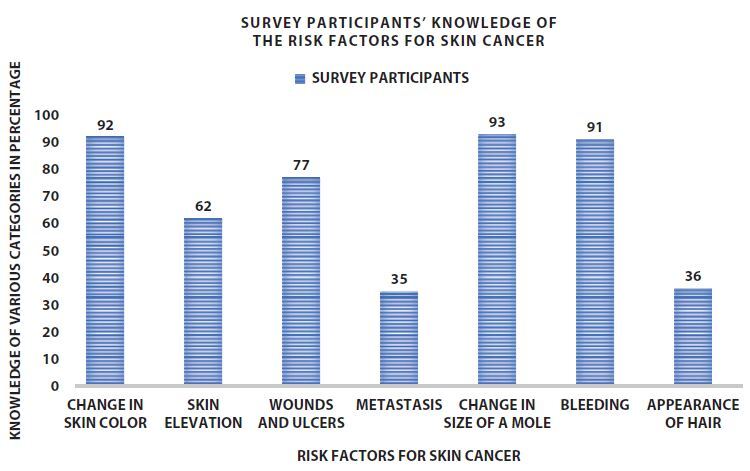
Clustered column showing survey participants’ knowledge of the risk factors for skin cancer.

Knowledge of the appearance of skin cancer was more common, with 92% (n=402, the number of respondents=437) of participants correctly reporting a change in skin color, while 62% (n=218, the number of respondents=352) correctly reported skin elevation, and 77% (n=308, the number of respondents=400) correctly reported wounds and ulcers. Only 35% (n=179, the number of respondents=511) of participants reported knowledge of metastasis, while 57% (n=295) reported no knowledge about skin cancer spread. The majority of participants correctly identified the most important risk factors for skin cancer (Figure 1).

Most participants had some knowledge of the indicators of cancer growth. Of 453 respondents, 93% reported change in size as an indication of cancer growth, while 88% reported change in color and 91% reported bleeding as important indicators. However, appearance of the hair was considered an important indication by only 36% of participants.

**Figure 2:**
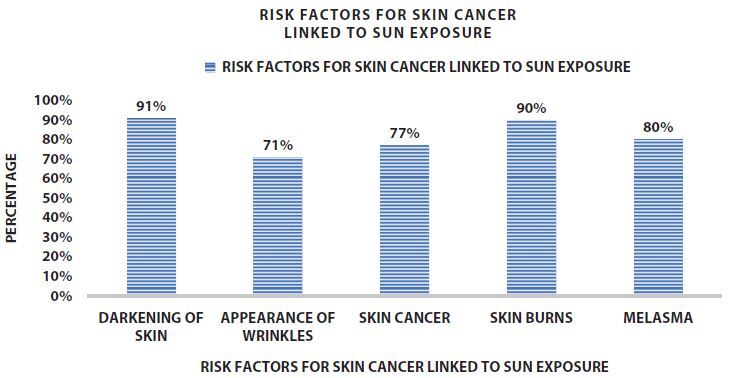
Clustered column showing participants’ understanding of the harmful effects of sunlight.

**Figure 3:**
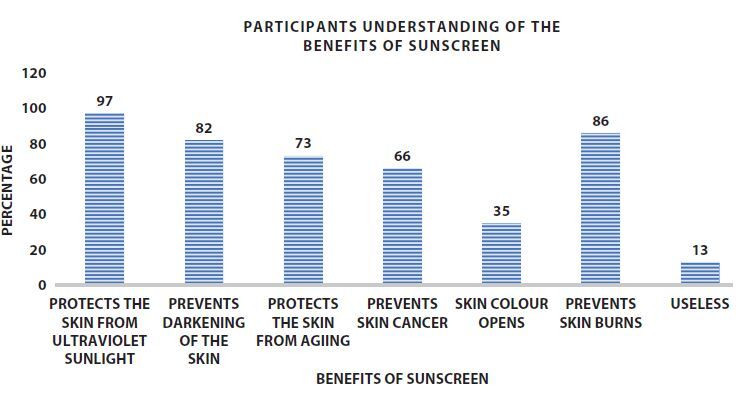
Clustered column showing participants’ understanding of the benefits of sunscreen.

More than half (54%) reported that wearing clothes to cover the skin was important for skin cancer prevention, while sunscreen use and periodic examination were considered significant by, respectively, 445 (90.4%, respondents= 492) and 417 (85.8%, respondents=486) participants. The majority of participants also correctly identified risk factors for skin cancer linked to sun exposure (Figure 2) and reported the benefits of sunscreen (Figure 3).

Less than half of participants reported some harmful effect of using sunscreen (Table 2). Most participants correctly reported that sunscreens differ from each other, and most reported at least some sunscreen use (Table 2). Participants reported that price (62%), density (66%), ingredients (75%), sun protection factor (SPF) (81%), doctor recommendation (82%) and country of the manufacturer (53%) were important factors behind their selection of a sunscreen. The primary reason that most participants reported as being behind a decision not to use sunscreen was time (56%). A minority (16%) reported complications of sunscreen use.

## Discussion

The findings of the study show varying awareness among people in Saudi Arabia on the subject of skin cancer. While the large majority of study participants correctly identified risk factors and preventative measures, most reported using sunscreen daily.

This study has multiple methodological issues. First, the large majority of participants were women, so data on the knowledge and behavior of Saudi men may be less reflective of the population. Second, although the study included participants from multiple geographic locations, the sample size was relatively small. Similar studies in other geographic areas and other countries may have different findings and limiting generalizability of the results. Last, in a cross-sectional study, causality cannot be ascertained. All data were self-reported, introducing a chance of recall bias. This manuscript reports on a convenience sample, representing the population of the Saudi Arabia to the extent possible.

However, this study also has several strengths. The use of a validated tool for measuring awareness provided a wider range of more specific results. Moreover, pairing assessment of a population’s knowledge with behavior provided information on what specific areas of information should be addressed.

The results show a much greater awareness of skin cancer in the general population of Saudi Arabia than a 2016 study conducted in Riyadh, in which 83% of participants did not know that sun exposure causes skin cancer [23], as well as a 2010 study in the Qassim Province, in which only 56% of participants had knowledge of the association [25]. The level of awareness reported in this study is similar to the results of a 2020 study in Jeddah, in which 73% of participants reported awareness of the association between skin cancer and sun exposure [24]. Sunscreen usage was inadequate in that study, with only 36% of participants using it regularly; this rate is slightly higher than that of other Saudi studies, in which only 24% of respondents reported regularly using sunscreen [26,27]. One possible reason for the relatively higher levels of awareness among participants in the present study is that 89% were women, and a previous study indicated that women are more likely to be knowledgeable about skin cancer than men [23]. However, the higher level of education in this study may also have made the awareness of skin cancer somewhat higher.

One important implication of this study is that, while knowledge about skin cancer and its risk factors may have increased, the practice of sun-protective behaviors continues to be alarmingly low. This finding indicates that the Saudi population may benefit from an increase in awareness campaigns targeting sunscreen usage as well as increased recommendations from medical professionals. Additionally, regular and affordable testing for skin cancer should be made available nationally, to increase both awareness of skin cancer as well as opportunities for early detection.

## Conclusions

General skin cancer knowledge appears to be higher among the Saudi population than in previous years, with social media being the most common source of information. However, sun-preventative behaviors, especially sunscreen use, continue to be inadequate, despite the high levels of awareness. Future studies should address more specific knowledge of skin cancer and skin protection behaviors using more participants from a broader sample of the population.

## Figures and Tables

**Table 1:** Sociodemographic Characteristics of the Study Participants (n=529)

Sociodemographic Characteristic		No. (%)
Sex		
	Male	58 (11.2)
	Female	459 (88.8)
	Missing data	12
Education		
	Secondary or below	67 (13.0)
	University	449 (87.0)
	Missing data	13
Information about skin cancer		
	Yes	285 (55.1)
	No	213 (41.2)
	Don’t know	19 (3.7)
	Missing data	12
Source of information		
	Audiovisual	110 (24.7)
	Print media	90 (20.2)
	Social media	189 (42.4)
	Relatives	57 (12.8)
	Missing data	83
Types of skin cancer		
	Yes	285 (55.4)
	No	3 (0.6)
	Don’t know	226 (44.0)
	Missing data	15

**Table 2:** Knowledge of Sunscreen Among study Participants (n=529)

Information on Sunscreen	No. (%)

Harm of using sunscreen	

Pimples	212 (47.7)
Missing data	85

Skin irritation	186 (42.2)

Missing data	88

Difference in sunscreen	

Yes	391 (75.)
Missing data	12

Usage of sunscreen	

Yes	418 (82)
Missing data	19

Use of sunscreen/day	

Once	329 (78.7)

Twice	67 (16.0)

Thrice	22 (5.3)

Application of sunscreen	

Face	462 (96.9)
Missing data	52

Hands	345 (77.7)
Missing data	85

Arms	103 (27.9)
Missing data	160

Other parts	57 (15.7)
Missing data	166
